# An amazing new *Capsicum* (Solanaceae) species from the Andean-Amazonian Piedmont

**DOI:** 10.3897/phytokeys.167.57751

**Published:** 2020-11-20

**Authors:** Gloria E. Barboza, Carolina Carrizo García, Marisel Scaldaferro, Lynn Bohs

**Affiliations:** 1 Instituto Multidisciplinario de Biología Vegetal (IMBIV), CONICET, Córdoba, Argentina & Facultad de Ciencias Químicas, Universidad Nacional de Córdoba, Córdoba, Argentina; 2 Instituto Multidisciplinario de Biología Vegetal (IMBIV), CONICET, Córdoba, Argentina & Department of Botany and Biodiversity Research, University of Vienna, Vienna, Austria; 3 Instituto Multidisciplinario de Biología Vegetal (IMBIV), CONICET, Córdoba, Argentina & Facultad de Ciencias Exactas, Físicas y Naturales, Universidad Nacional de Córdoba, Córdoba, Argentina; 4 School of Biological Sciences, University of Utah, Salt Lake City, UT, USA

**Keywords:** Andean clade, *
Capsicum
*, chromosomes, phylogeny, South America, taxonomy

## Abstract

*Capsicum regale* Barboza & Bohs, **sp. nov.**, a new species from the tropical wet forests of the eastern Andean slopes (Colombia, Ecuador, and Peru) is described and illustrated. This new species belongs to the Andean clade (all species 2n = 26) of *Capsicum* and is similar to *C. longifolium* Barboza & S.Leiva in its glabrescence, calyx morphology, and corolla and seed color but differs in its membranous and elliptic leaves, fleshy calyces, deeper stellate corollas, longer filaments, longer and purple fruiting pedicels, purple berries, and larger seeds. Its chromosome number was counted (2n = 26), a preliminary assessment of conservation status is given and discussed, and an updated identification key to the species of the Andean clade is provided.

## Introduction

*Capsicum* L. (Capsiceae, Solanaceae), the chili pepper genus, consists of approximately 42 species distributed in temperate and tropical Central and South America, Mexico and the West Indies (Barboza et al. 2020). It includes five species cultivated worldwide as vegetables, spices, and medicines (*C. annuum* L., *C. frutescens* L., *C. chinense* Jacq., *C. baccatum* L. and *C. pubescens* Ruiz & Pav.). *Capsicum* peppers are major crops worldwide, and along with potato, tomato, and eggplant in the genus *Solanum* L., are amongst the most economically important members of the Solanaceae (Samuels 2015).

The Andes are one of the main centers of diversity for *Capsicum*, where new species continue to be discovered (Nee et al. 2006; Barboza et al. 2019). Approximately 50% of the species (ca. 20 species) occur in tropical Andean forests or in dry inter-Andean valleys (Barboza et al. in prep.). The tropical montane forest ecoregion is located on the slopes of the Andes extending north to south from southern Colombia, through Ecuador, and into northern Peru (WWF 2020). This region is characterized by a lush vegetation with evergreen seasonal broad-leaved forests and a rich fauna (Stewart et al. 2020; WWF 2020). It is one of the most biologically diverse ecosystems in the world (Gentry 1992; Bruijnzeel et al. 2010; Tapia-Armijos et al. 2015) with a high level of species endemism (Myers et al. 2000). Khoury et al. (2020) have demonstrated that the highlands of Colombia, Ecuador, Peru, and Venezuela represent one of the hotspots for *Capsicum* that need further investigation in terms of collecting taxa for *ex situ* conservation of the wild species.

During recent field explorations in the Colombian Cordillera Oriental (Dept. Caquetá), an atypical species of Solanaceae was collected. Despite the presence of several Solanaceae experts in the group, no one was sure what genus it belonged to. Its deeply stellate yellowish corollas, long-exserted stamens, and purple fruits and fruiting pedicels were striking and called to mind some characters of the poorly known genus *Cuatresia* Hunz., whereas its thick, triangular-compressed, and reflexed calyx appendages resembled those of some *Lycianthes* (Dunal) Hassler taxa, whose species are not well understood in Colombia. Puzzled, we provisionally named it “*Cuatresianthes*” and placed some bets on its eventual generic identity. DNA was extracted and sequenced in the Bohs lab from leaf material collected on these field trips. BLAST results indicated that the species belonged not to *Cuatresia* or *Lycianthes*, but to *Capsicum*. A preliminary molecular study placed the collection unequivocally in the Andean clade of *Capsicum*, but it did not belong to any known species. Through an exhaustive search amongst unidentified *Cuatresia* collections in herbaria, we found other specimens from Ecuador and Peru that matched our Colombian gatherings. Here, we describe this species as new to science and provide information on its morphology, distribution, karyology and phylogenetic position in the genus *Capsicum*.

## Materials and methods

Two field trips were made in Colombia (Dept. Caquetá) during 2016 and 2019. Fresh material was preserved in 70% alcohol to perform measurements of reproductive organs using a Zeiss Stemi 2000-C stereomicroscope at 6.5–50× magnification. Descriptions were based on living plants observed during field work and examination of digital images of herbarium specimens housed at the following seven herbaria: BM, COAH, COL, F, MO, QCNE, US. Seeds were also examined using scanning electron microscopy (SEM); they were prepared using enzyme etching (Lester and Durrands 1984) to dissolve outer cell walls, affixed to aluminum stubs with double-sided adhesive tape, coated with gold, and examined using a FE-SEM Sigma (LAMARX, National University of Córdoba, Argentina) microscope.

Information about flower, fruit, and seed color was taken mainly from our own observations in the field and photographs sent by some collectors; we tested pungency in the field on immature and mature fruits.

The distribution map was produced using QGIS 3.8 (QGIS Development Team 2019) and was based on georeferenced data of all the collections analyzed. Conservation status was assessed using IUCN criteria B, geographic range in the form of B1 (EOO: extent of occurrence) and B2 (AOO; area of occupancy) (IUCN 2019). The extent of occurrence and area of occupancy were calculated using the Geospatial Conservation Assessment Tool GeoCAT (Bachman et al. 2011; GeoCAT 2020).

Somatic metaphases were examined in root tip squashes obtained from germinated seeds. The root apices were fixed in 3:1 ethanol: acetic acid mixture for 12 hr after a pretreatment in 2 mM 8-hydroxyquinoline solution for two hr at room temperature and two hr at 4 °C. The material was kept at –20 °C until examination. The root tips were macerated in pectinase-cellulase solution (Moscone et al. 1993), and chromosomes were stained with 4’–6-diamidino-2-phenylindole (DAPI) (Schweizer and Ambros 1994). Metaphase chromosomes were observed and photographed with epifluorescence using an Olympus BX61 microscope equipped with the appropriate filter sets (Olympus, Shinjuku-ku, Tokyo, Japan) and a JAI CV-M4 + CL monochromatic digital camera (JAI, Barrington, N.J., USA). Three individual seeds from the collection *Orejuela et al. 3034* were germinated and grown until root tips were produced, and 10 cells from each seedling were studied in metaphases.

Phylogenetic affinities were explored using DNA sequences from four markers, namely: the intergenic spacers *psb*A-*trn*H, *ndh*F-*rpl*32 and *trn*L-*trn*F from the plastid genome, and the single-copy nuclear gene *waxy* (GBSSI, granule-bound starch synthase, exons 2 to 10). Representatives of different clades recognized within *Capsicum* and several outgroup species were included. Genomic DNA of *C. regale* was extracted from silica-gel dried leaves using the Qiagen DNeasy Plant mini kit (Qiagen Inc., Valencia, California, EUA) and a modified CTAB protocol. Most sequences included in this study were used in previously published analyses and therefore were retrieved from GenBank, except for a few sequences from outgroup species (see Suppl. material 1: Table S1), for which DNA extracts were already available. Amplification and sequencing protocols for the markers used were as in Carrizo García et al. (2016, 2020) and Barboza et al. (2019). PCR amplicons were sequenced on an automated capillary sequencer [University of Vienna (Vienna, Austria), and the University of Utah HSC Core Research Facility (Salt Lake City, Utah, USA)]. A single concatenated dataset was assembled in MEGA 7 (Kumar et al. 2016). Phylogenetic reconstructions were done using maximum parsimony [MP, in PAUP* 4.0b10 (Swofford 2003)], maximum likelihood [ML, in RAxML v8.2.10 (Stamatakis 2014)] and Bayesian inference [BI, in MrBayes 3.2.2 (Ronquist et al. 2012)] approaches as in Carrizo García et al. (2016, 2020). The GTR+R nucleotide substitution model was selected a priori following the Akaike Information Criteria in jModelTest 2.1.3 (Darriba et al. 2012) for ML and BI analyses.

## Taxonomic treatment

### Capsicum regale

Taxon classificationPlantaeSolanalesSolanaceae

Barboza & Bohs sp. nov.

4946A53F-66C3-5E60-AE2F-4370E1AE79A0

urn:lsid:ipni.org:names:77212951-1

Figs 1 , 2 , 3

#### Diagnosis.

*Capsicum regale* is morphologically most similar to *C. longifolium* Barboza & S.Leiva, but the former differs in having membranous and elliptic leaves, fleshy calyces, more deeply stellate corollas, longer filaments, longer and purple fruiting pedicels, dark blue to purple berries, larger seeds, smooth seed coats, and spine-like projections along the seed margins.

**Figure 1.**
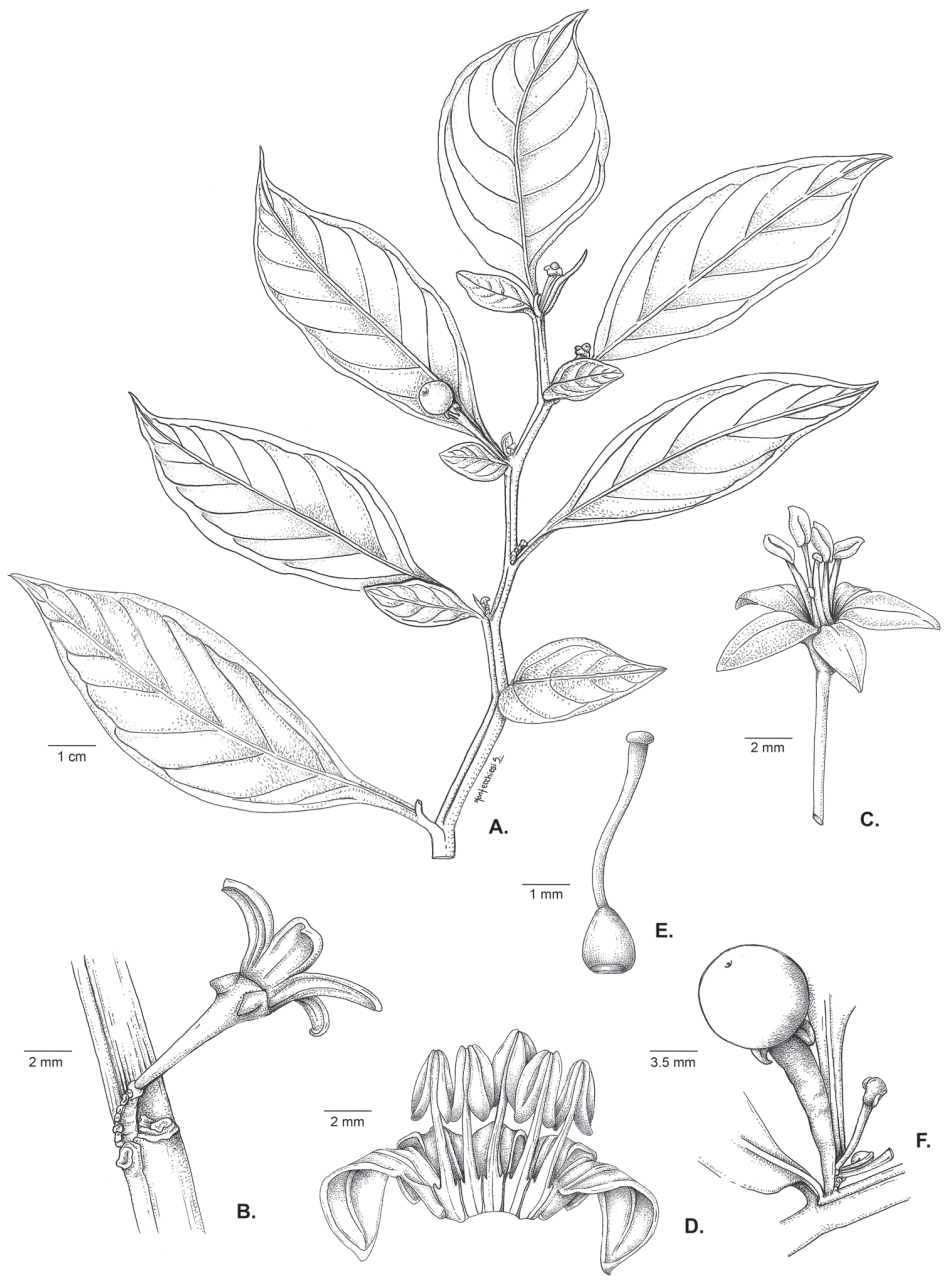
*Capsicum regale* Barboza & Bohs. **A** fruiting apical branch **B** unbranched inflorescence **C** flower, in lateral view **D** opened corolla **E** gynoecium **F** fruit. From *Orejuela et al. 3034*. Drawn by S. Montecchiesi.

#### Type.

Colombia. Caquetá: Mun. Florencia, Corregimiento El Caraño, Finca de Don Isauro, camino al río, en interior de bosque fuertemente inclinado, 01°44'10.6"N, 75°40'78.3"W, 1004 m, 22 Aug 2019 (fl, fr), *A. Orejuela, L. Bohs, G.E. Barboza, P. González, R. Deanna, J. Urdampilleta, J. Valencia & G. Sierra 3034* (holotype: COL; isotypes: COAH, CORD, HUAZ [to be distributed]).

**Figure 2.**
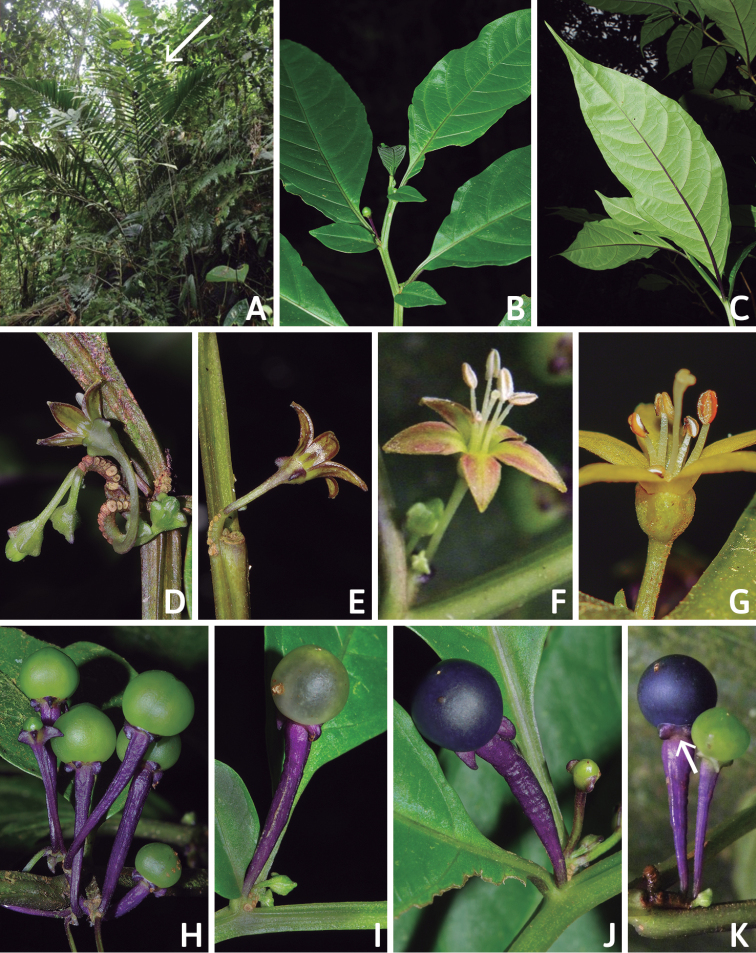
*Capsicum regale* Barboza & Bohs. **A** habitat **B** apical branch, showing anisophyllous leaf pairs **C** abaxial surface of leaf with purple main vein **D** forked inflorescence; note the scars of the deciduous flowers **E** flower, in lateral view, on a unbranched elongate inflorescence **F, G** Flowers with and without pigmentation respectively **H–K** various stages of fruit maturity, in K mature fruit showing the constriction between the pedicel and the berry (arrow) **A–F, H–K** from *Orejuela et al. 3034* (photos by A. Orejuela, P. Gonzáles, and G. Barboza) **G** from *Hoyos 127* (photo by L. Coca).

#### Description.

Slender shrubs (1–) 1.8–2.5 (–3) m tall, with the main stem somewhat thick, ca. 0.8 cm in diameter at base, sparsely branched toward apex, the branches dichotomous, weak, spreading horizontally. Stems solid and terete at base, the young stems pale green, glossy, striate, glabrous, the nodes green; bark of older stems dark brown, glabrous; lenticels present. Sympodial units difoliate, geminate, the leaf pairs markedly differing in size. Leaves simple, membranous, slightly discolorous, green adaxially, pale green with the midvein prominent and purple and the secondary veins lilac or green abaxially; adaxial and abaxial surfaces glabrous; major leaves with blades 17–20 (–24) cm long, 4.7–8 (–9.2) cm wide, elliptic, the major veins 6–8 on each side of midvein, the base unequal and attenuate, the margins entire and glabrous, the apex apiculate to long-apiculate; petioles (0.8–) 1.5–2.3 cm long, green adaxially and purple abaxially, glabrous; the minor leaves 2–5 cm long, 1–3 cm wide, ovate, the major veins 3–5 on each side of midvein, the base unequal, the margins entire, glabrous, the apex obtuse; petioles 0–0.4 cm long, green, glabrous. Inflorescence ca. 10 mm long, unbranched or rarely shortly forked, with 5–13 flowers, the axes glabrous; peduncle 0–5.5 mm; rachis 4.5–6 mm long; pedicels 1.2–1.4 cm long, thin, 2–3–edged, erect to spreading, straight, purple to green, glabrous, nearly contiguous, articulated at the base, leaving conspicuous scars. Buds ellipsoid, green. Flowers 5-merous, all perfect. Calyx 2–3 mm long, ca. 2 mm wide, cup-shaped, fleshy, green or greenish purple, the margin truncate, circular in outline, glabrous, the appendages (0–) 4–5, 1–1.8 mm long, 0.8–1.1 mm wide, purple, thick, triangular-compressed, reflexed, inserted very close to the margin. Corolla 7–8 mm long, ca. 10 mm in diameter, deeply stellate, thick, with narrow interpetalar tissue, pure yellow or yellow with maroon pigmentation abaxially and greenish yellow with lobes marginally maroon adaxially, glabrous, the tube 2–2.5 mm long, the lobes 5–5.5 mm long, ca. 2 mm wide, triangular, the tips papillose, the margins with short eglandular trichomes. Stamens subequal, one filament longer than the others; long filament 3.5–4.3 mm long, shorter filaments (2) 3–3.2 mm long, white, glabrous, inserted on the corolla ca. 1 mm from the base, with inconspicuous auricles; anthers ca. 2 mm long, elliptic, not connivent, the thecae lilac or pale bluish, opening into longitudinal slits. Ovary ca. 1.3 mm long, ca. 1 mm in diameter, light green, ovoid, glabrous; nectary ca. 0.4 mm high, paler than the ovary, conspicuous; style 4.3–4.5 mm long, white, clavate, glabrous; stigma ca. 0.1 mm long, ca. 0.8 mm wide, light green, globose or somewhat discoid. Fruit a berry, globose, 6–9 mm in diameter, green when immature, turning nearly white and translucent during transition to maturity, then becoming dark blue to purple when mature, glabrous, non-pungent, the pericarp opaque, without giant cells, the endocarp smooth; stone cells absent; fruiting pedicels ca. 1.8 cm long, 1.8–2 mm in diameter proximally, 2.5–2.6 mm in diameter distally, brilliant dark purple, erect, fleshy, slightly angled and strongly thickened distally; fruiting calyx 3.75–4.25 mm in diameter, persistent, not accrescent, discoid, brilliant purple, with a conspicuous annular constriction at the junction with the swollen pedicel, the appendages reflexed, brilliant purple, fleshy and laterally compressed. Seeds 7–17 per fruit, 2.7–3.4 mm long, 2.2–2.7 mm wide, flattened, C-shaped, black, the seed coat smooth except for small spine-like projections on the seed margin, the cells irregular in shape to polygonal at seed margins, the lateral walls sinuate to straight.

**Figure 3.**
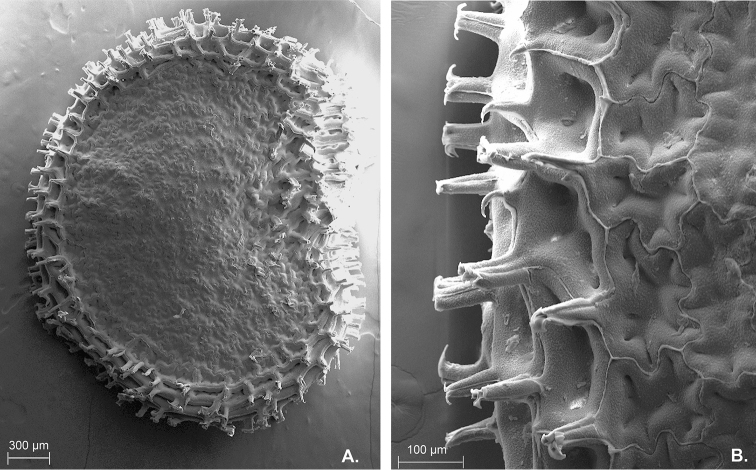
Seed of *C. regale* Barboza & Bohs viewed under SEM. A Seed B Seed coat sculpture. From *Orejuela et al. 3034*.

#### Distribution.

**Figure 4.**
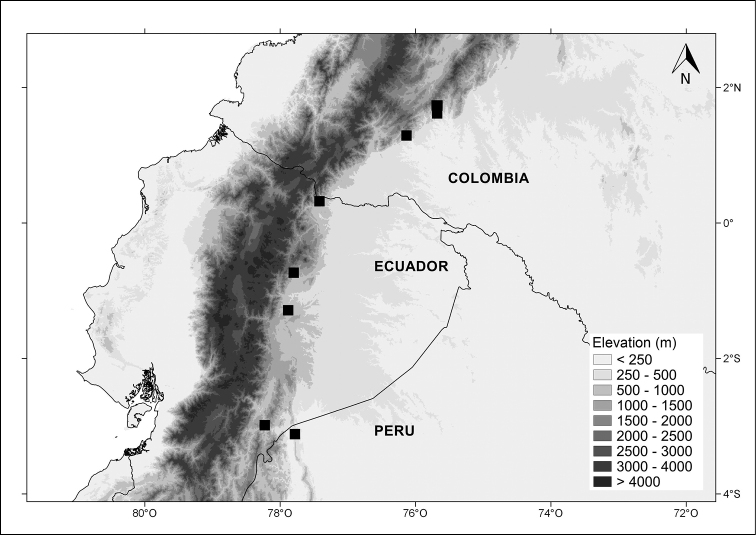
Distribution of *Capsicum regale* Barboza & Bohs.

*Capsicum regale* occurs in southern Colombia, eastern Ecuador, and northern Peru, known mainly on the eastern slopes of the Andes (the Andean-Amazonian Piedmont), between 700–1900 m elevation (Fig. 4).

#### Ecology.

The small populations inhabit the understory of the premontane or montane humid tropical forests of the Amazonian slopes of the Andes.

#### Phenology.

The species has been collected in flower and fruit in April and from August to December.

#### Etymology.

The specific epithet comes from the Latin *regalis*, royal or regal, referring to the regal, princely, or magnificent appearance of this special plant and also making reference to the royal purple color that suffuses the leaves, fruits, and fruiting pedicels.

#### Preliminary assessment of conservation status.

Assessment using the IUCN Red List Criteria (IUCN 2019) suggests a status of Endangered (EN) B2ab(iii) for *C. regale*. Although this species has an extent of occurrence (EOO) of 47,806.378 km^2^, its area of occupancy (AOO) is calculated to be 32 km^2^ (criterion B2 < 500 km^2^), and the habitat quality has experienced a continuing decline, especially associated with fragmentation and deforestation.

#### Chromosome number.

**Figure 5.**
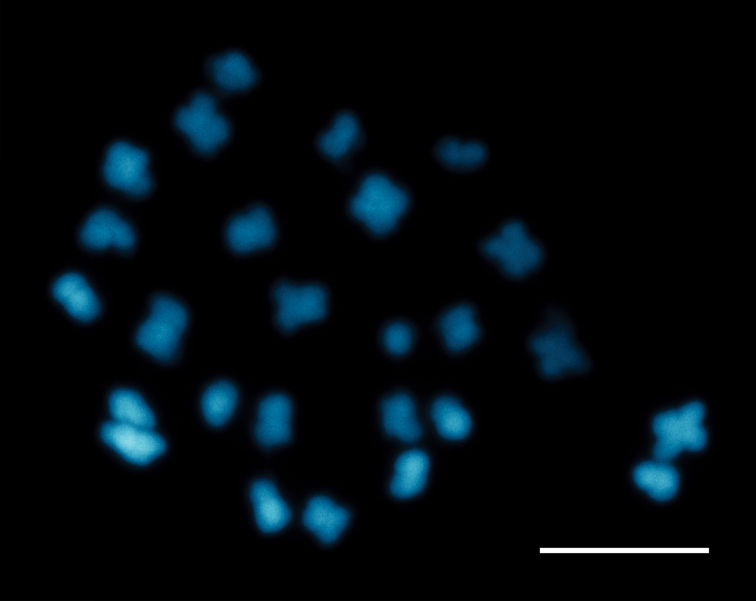
Mitotic metaphase chromosomes of *Capsicum regale* Barboza & Bohs, 2n = 26. Scale bar: 10 µm

The somatic chromosome number found in *C. regale* is 2n = 2x = 26 (Fig. 5), as for all of the species of the Andean clade (Scaldaferro and Moscone 2019; Barboza et al. 2019).

#### Phylogenetic affinities.

**Figure 6.**
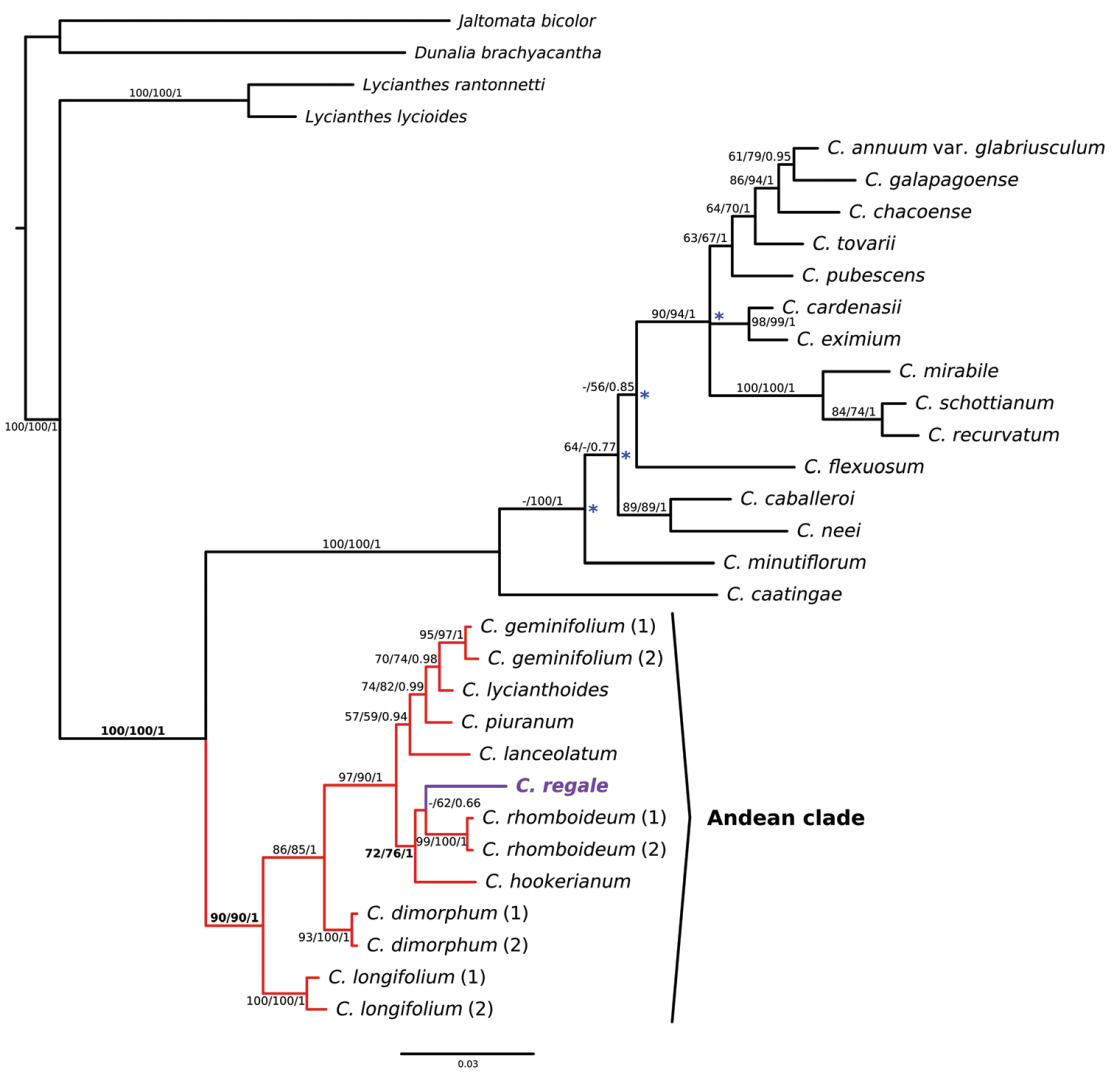
Bayesian majority-rule consensus tree of *Capsicum* showing the placement of *C. regale* Barboza & Bohs. The Andean clade is highlighted in colored branches. Support values are indicated by each branch (bootstrap support maximum parsimony/bootstrap support maximum likelihood/posterior probabilities; dashes indicate support values < 50%). Key support values that indicate the position of *C. regale* are shown in bold. Asterisks indicate different resolutions using maximum parsimony.

*Capsicum regale* is strongly resolved within the Andean clade of *Capsicum* in all analyses. Within the Andean clade, *C. regale* is moderately supported in a clade with *C. rhomboideum* and *C. hookerianum*. Within this clade, it is weakly supported as sister to *C. rhomboideum* (Fig. 6).

#### Specimens examined.

Colombia. Caquetá: Mun. Florencia, Corregimiento El Caraño, Km 20, finca Las Brisas, propiedad de Isauro Trujillo, 01°44'11.80"N, 75°40'37.8"W, 1002 m, 7 Oct 2017 (fl, fr), *D. Hoyos, E. Trujillo & J. Sánchez 118* (COAH, COL); same locality, 9 Dec 2017 (fl, fr), *D. Hoyos, M. Cuellar & F. Vallejo* 146 (COL); Finca de don Isauro, camino al río, en interior de bosque fuertemente inclinado, 01°44'01.4"N, 75°40'35.4"W, 1000 m, 16 Apr 2016 (fl, fr), *A. Orejuela, L. Bohs, G.E. Barboza, E. Trujillo, J. D. Tovar & J. Castillo 2640* (COL); same locality, 01°44'09.1"N, 75°40'40.3"W, 932 m, 22 Aug 2019 (fl, fr), *A. Orejuela, L. Bohs, G.E. Barboza, P. González, R. Deanna, J. Urdampilleta, J. Valencia & G. Sierra 3035* (COL); finca Las Brisas, debajo de la casa, vereda La Cascada, 01°37'5"N, 75°40'50"W, 1000 m, 7 Nov 2015 (fl, fr), *D. Sanín 6236* (COL); Mun. San José del Fragua, vereda La Peneya-camino hacia El Jardín, zona amortiguadora PNN Alto Fragua Indi Wasi, 01°17'31"N, 76°08'0.64"W, 700–850 m, 23 Oct 2017 (fl, fr), *D. Hoyos et al. 127* (COAH, COL).

ECUADOR. Morona-Santiago: along new road Mendez-Morona, km 30–35, 800 m, 18 Aug 1989 (fl, fr), *H. van der Werff & E. Gudiño 11196* (BM, MO, QCNE). Napo: Archidona Cantón, Reserva Ecológica Antisana, Comunidad Shamato, entrada por km 21-Shamato, 00°44’S, 77°48’W, 1700 m, 27 Apr 1998 (fl), *J. L. Clark et al. 5337* (BM, MO); Parroquia Ahuano, Estación Biológica Jatun Sacha, 8 km E of Misahuallí, Finca Acaro, 01°17'17"S, 77°52'54"W, 910 m, 17 Aug 2005 (fl, fr), *J. L. Clark et al. 9403* (BM, US). Sucumbíos: Río Bermejo to Cerro Sur Pax, Cofan community of Alto Bermejo, NW between Lumbaqui and Cascales, vicinity of Oso Ridge Camp, 00°19'17.7"N, 77°25'10"W, 1700–1920 m, 2 Aug 2001 (fr), *R. Aguinda et al. 1537* (F).

PERU. Loreto: Datem del Marañón, Morona District, Pongo Chinim, valley between the eastern and western ridges of the Kampankis range, ca.14 km south of the Peru-Ecuador border, 3 Aug 2011 (fl, fr), *I. Huamantupa 15251* (V0387079F color photo, F).

## Discussion

*Capsicum regale* belongs to the Andean clade of *Capsicum* (Carrizo García et al. 2016; see below). It is a very striking species due to its unbranched (Figs 1B, 2E, J) or forked inflorescence (Fig. 2D) with 5–13 deciduous flowers on an elongate rachis (Fig. 2D), fleshy and laterally compressed calyx appendages (Fig. 2D, E), deeply stellate corollas (Fig. 2F, G), strongly thickened and brilliant purple fruiting pedicels (Fig. 2H–K), dark blue to purple fruits (Fig. 2J, K), and flattened black seeds with spine-like projections at the margins (Fig. 3). This species is morphologically most similar to *C. longifolium* (Barboza et al. 2019) with which it shares lack of pubescence, multi-flowered inflorescences, yellow corollas, laterally compressed calyx appendages, and black seeds (see contrasting characters in the key below).

*Capsicum regale* possesses unusual characters of the genus. Normally, *Capsicum* species have unbranched inflorescences lacking peduncles, with the flowers solitary or congested on a very short axis. Flowers can be arranged on a short or relatively elongated rachis in a few species, e.g., *C. rhomboideum* (Dunal) Kuntze, *C. coccineum* (Rusby) Hunz., *C. lycianthoides* Bitter (Barboza pers. obs.), *C. longifolium* (Barboza et al. 2019), and *C. regale*, but none of them have short peduncles or forked inflorescences as occurs occasionally in *C. regale*. In most *Capsicum* species the calyx appendages, when present, are usually cylindrical or subulate, and green-colored. It is very rare to find laterally compressed calyx appendages that appear as wing-like structures, as occur in *C. longifolium* (Barboza et al. 2019), in some plants of *C. dimorphum* (Miers) Kuntze (Barboza, pers. obs.), and in *C. regale*. Stellate corollas lobed about halfway to the base are common in the genus; exceptions to this are found in *C. benoistii* Barboza (Barboza et al. 2019) and *C. regale*, both of which have deeply stellate corollas lobed more than halfway to the base. In most *Capsicum* species, the fruiting pedicels and fruiting calyx are generally green or green with purple tones or lines; only *C. caatingae* Barboza & Agra (Carrizo García et al. 2016) and sometimes *C. dimorphum* and *C. geminifolium* (Dammer) Hunz. (Jarret et al. 2019) have pedicels and calyces uniformly violet-colored, while those of *C. regale* are uniformly purple-colored. An unusual constriction at the junction of the thickened fruiting pedicels with the fruiting calyx is clearly evident in *C. regale* (Fig. 2K), a character also present in some other species, i.e., *C. chinense* Jacq. (Baral and Bosland 2004), *C. caatingae* (Carrizo García et al. 2013), *C. minutiflorum* (Rusby) Hunz. (Carrizo García et al. 2016), and *C. lanceolatum* (Greenm.) C.V. Morton & Standl. (Barboza pers. obs.). The dark blue to purple fruits are unique to *C. regale* among the wild *Capsicum* species, which have red, orange-red, or greenish-golden yellow fruits at maturity (Hunziker 2001; Carrizo García et al. 2016).

Carrizo García et al. (2016) were the first to provide an extensive phylogenetic analysis of *Capsicum* using broad sampling of 34 of the approximately 35 species of the genus known at the time. They identified and named 11 well supported clades within *Capsicum*. One of these is the Andean clade, which includes species native to Central America and the Andes in northwestern South America. Morphological characters of the Andean clade species include leaves borne in anisophyllous pairs, flowering pedicels straight (not geniculate), corollas mainly yellow, fruits red to orange-red and non-pungent with the pericarp lacking giant cells, seeds black or blackish-brown, and chromosome base numbers of x = 13 (Jarret et al. 2019; Scaldaferro and Moscone 2019). *Capsicum regale* exhibits all of these characters except for its dark blue or purple fruits and the occasional forked inflorescences, which are not known in any other wild *Capsicum* species. Molecular data from both chloroplast and nuclear regions place *Capsicum regale* within the Andean clade (Fig. 6). Although *C. regale* is morphologically most similar to *C. longifolium*, the combined molecular data places it in a clade with *C. rhomboideum* and *C. hookerianum* with moderate support. Nevertheless, its closest specific affinities need to be further studied using additional data.

*Capsicum regale* inhabits the Andean-Amazonian Piedmont, encompassing the eastern slopes of the Cordillera Oriental from southern Colombia to the Cerros de Kampanquis, the easternmost branch of the Andes in northern Peru. This area is home to a transitional ecosystem with a distinctive vegetation and biodiversity due, in part, to the juxtaposition between the Amazon basin and the Andean forests (Gentry 1992; Pitman et al. 2002); this unique biodiversity is rapidly disappearing due to intense deforestation, clearing, and fragmentation (Pitman et al. 2002; Mulligan 2010; Tapia-Armijos et al. 2015; Alvarez-B et al. 2019). Some localities where *C. regale* has been collected are protected areas (Parque Nacional Natural Alto Fragua Indi Wasi, Colombia; Reserva Ecológica Antisana, Ecuador; Estación Biológica Jatun Sacha, Ecuador), and it is expected that in these reserves it is not under serious threat. Other sites in which it occurs are susceptible to human disturbance such as crop planting and high levels of deforestation; these locations include Correg. Caraño (Caquetá, Colombia, Alvarez-B et al. 2019, our observations), Río Bermejo to Cerro Sur Pax (Sucumbíos, Ecuador, Pitman et al. 2002), and Cuenca del Río Morona, Pongo Chinim (Loreto, Peru, Pitman et al. 2012). In these areas, *C. regale* is considerably threatened, and a conservation strategy is urgently needed to protect these species-rich ecoregions.

### Artificial key to the species of Andean clade of *Capsicum*

**Table:** 

1	Flowers solitary, rarely paired; pedicels (15–) 25–43 mm long; calyx with 5 subequal reflexed appendages; corolla white or yellowish-white lined with purple; Mesoamerica	***C. lanceolatum* (Greenm.) C.V.Morton & Standl.**
–	Flowers 2–10 (–13), rarely solitary; pedicels 3–28 mm long; calyx lacking appendages or with up to 10 subequal or unequal, recurved, spreading or erect appendages; corolla pure yellow or yellowish with maroon or purple pigmentation; South America (*C. rhomboideum* also in Mesoamerica)	**2**
2	Calyx appendages absent, or appearing as 1–3 small 0.5–1.8 mm long mucronate protuberances below the margin, or well-developed, 2–5, triangular-compressed and wing-like, 2–2.5 mm long	**3**
–	Calyx appendages (2–) 5–10, subulate or linear-subulate, (0.9–) 2–7 mm long	**5**
3	Plants usually pubescent, rarely glabrescent; flowers up to 5, axillary, the rachis very reduced or lacking; calyx with 0–3 small mucronate appendages 0.5–1.5 mm long	***C. dimorphum* (Miers) Kuntze**
–	Plants completely glabrous; flowers 3–13, on a developed rachis; calyx with 2–5 thick triangular-compressed wing-like appendages 1–2.5 mm long	**4**
4	Leaves coriaceous; major leaves narrowly elliptic (ratio length/width 6–10.8); corolla stellate-campanulate, lobed about halfway to base; calyx tube membranous; stamens equal, 2–2.6 mm long; fruits 8–13 mm in diameter, orange at maturity; fruiting pedicels 1–1.6 cm long, green, pendent; fruiting calyx green-purple or green; seeds 1.7–2.3 mm long, 1.7–2.2 mm wide, not flattened, tear drop-shaped, the surface reticulate	***C. longifolium* Barboza & S.Leiva**
–	Leaves membranous; major leaves elliptic (ratio length/width 2.5–4); corolla deeply stellate, lobed more than halfway to base; calyx tube fleshy; stamens subequal (one longer), (2–) 3–4.3 mm long; fruits 6–9 mm in diameter, dark blue to purple at maturity; fruiting pedicels ca. 1.8 cm long, brilliant dark purple, erect; fruiting calyx entirely brilliant purple; seeds 2.75–3.40 mm long, 2.25–2.70 mm wide, flattened, C-shaped, the surface smooth with small spine-like projections	***C. regale* Barboza & Bohs**
5	Calyx with 8–10 unequal appendages, the longer 4–6 (–7) mm long, the shorter 1.3–4 mm long	***C. hookerianum* (Miers) Kuntze**
–	Calyx with 2–5 equal or subequal appendages 0.9–6.5 mm long	**6**
6	Flowers 1–3, axillary; corolla long tubular-campanulate, 14.5–17 mm long, the tube 11–12 mm long, the lobes broadly ovate, 3.5–5 mm long, 4.5–5 mm wide; stone cells 2	***C. piuranum* Barboza & S.Leiva**
–	Flowers (2–) 3–10 (–13), axillary or on a short rachis; corolla deeply stellate or campanulate to broadly campanulate, (6–) 7–15 mm long, the tube 3–12 (–15) mm long, the lobes absent or incipient to well developed, narrowly triangular or ovate to broadly ovate, (3–) 5–9 mm long, 2–5.5 mm wide; stone cells absent or 5–6 (fruits unknown in *C. benoistii*)	**7**
7	Corolla deeply stellate, 12–13 mm long, the lobes narrowly triangular	***C. benoistii* Barboza**
–	Corolla nearly entire, campanulate to broadly campanulate, (6–) 7–15 mm long, the lobes absent or incipient, ovate to broadly ovate	**8**
8	Corolla campanulate, stellate in outline, with a thin interpetalar membrane connecting the lobes in the proximal half	***C. geminifolium* (Dammer) Hunz.**
–	Corolla broadly campanulate, pentagonal in outline, with a wide interpetalar membrane connecting the lobes up to the distal end	**9**
9	Inflorescence up to 13-flowered; major leaves membranous, (4–) 4.8–12 cm long, 2–5 cm wide, ovate, elliptic, or rhomboid-ovate; corolla 6–9.5 mm long, 8–12 mm in diameter; fruits up to 0.9 cm in diameter, dark red at maturity; stone cells absent; trees or erect shrubs; trichomes simple, branched, and dendritic on the same plant	***C. rhomboideum* (Dunal) Kuntze**
–	Inflorescences (2–) 3–8 (–10)-flowered; major leaves coriaceous, (10–) 11–22.5 cm long, (3–) 4–8.5 cm wide, ovate to broadly ovate; corolla 8–15 mm long, 15–18 mm in diameter; fruits up to 1.2 cm in diameter, bright orange or red at maturity; stone cells 0–6; scandent or slender shrub or subshrub; mostly glabrous or sparse, simple trichomes present on young stems only	***C. lycianthoides* Bitter**


## Supplementary Material

XML Treatment for Capsicum regale

## Supplementary materials

Supplementary material 1 Table S1. Taxa and materials analyzed in the phylogenetic analyses Data type Excel file Explanation note Taxa and materials analyzed in the phylogenetic analyses. Position within *Capsicum* (clade) or as outgroup, provenance, voucher specimens, ID in trees, and GenBank accession numbers for each marker analyzed are provided. Sequences retrieved from GenBank are marked with an asterisk. Abbreviations. CGN = Centre for Genetic Resources, Wageningen University, NL. NMCA = College of Agriculture, New Mexico State University, USA. Cult. = cultivated. Authors: Gloria E. Barboza, Carolina Carrizo García, Marisel Scaldaferro & Lynn Bohs. https://binary.pensoft.net/file/477459This dataset is made available under the Open Database License (http://opendatacommons.org/licenses/odbl/1.0/). The Open Database License (ODbL) is a license agreement intended to allow users to freely share, modify, and use this Dataset while maintaining this same freedom for others, provided that the original source and author(s) are credited.Gloria E. Barboza, Carolina Carrizo García, Marisel Scaldaferro, Lynn Bohs
